# Group-based trajectory modeling for supportive care needs in Chinese cancer survivors: A systematic review

**DOI:** 10.1016/j.apjon.2025.100738

**Published:** 2025-06-06

**Authors:** Kexin Li, Shi Chen, Ran Xu, Xiaohui Dong, Xianying Lu, Xinyu Chen, Chaoming Hou, Jing Gao

**Affiliations:** College of Nursing, Chengdu University of Traditional Chinese Medicine, Chengdu, China

**Keywords:** Supportive care needs, Cancer survivors, Group-based trajectory modeling, Influencing factors, Systematic review

## Abstract

**Objectives:**

This study aims to systematically synthesize studies that applied group-based trajectory modeling to examine the trajectories of supportive care needs (SCNs) among cancer survivors and to identify associated influencing factors.

**Methods:**

A comprehensive literature search was conducted in the following databases: CINAHL, Cochrane Library, Embase, PubMed, Web of Science, CNKI, SinoMed, VIP, and Wanfang. Studies were screened and assessed independently by two reviewers using the Joanna Briggs Institute (JBI) Critical Appraisal Checklist for Cohort Studies and the Guidelines for Reporting on Latent Trajectory Studies (GRoLTS) checklist. Key study characteristics and findings were extracted and synthesized narratively.

**Results:**

Ten studies met the inclusion criteria. Five distinct SCNs trajectories were identified across studies, including high-stable, moderate-stable, low-stable, decreasing, and increasing patterns. A total of 18 statistically significant influencing factors were identified and categorized according to the five domains of the Social-Ecological Model (SEM). Most factors were related to demographic characteristics, clinical variables, and individual symptoms.

**Conclusions:**

SCNs trajectories among cancer survivors exhibit substantial heterogeneity. While individual- and disease-level factors are commonly reported, limited evidence exists regarding the role of social support, healthcare system factors, and community-level influences. Future research should incorporate broader socio-environmental determinants to enhance understanding of SCNs patterns and inform tailored survivorship care.

**Systematic review registration:**

PROSPERO CRD42024586557.

## Introduction

Cancer has emerged as one of the most significant public health challenges globally, posing a substantial threat to human health and well-being.^1^ According to the latest data from the International Agency for Research on Cancer (IARC), there were close to 20 million new cases of cancer in the year 2022, and the number of new cases of cancer will reach more than 35 million by 2050 with demographics-based predictions.^2^ Although past evidence from the whole world demonstrated that the mortality of cancer remained high level,^3^ fortunately, evidences have demonstrated that the mortality of cancer has indicated a downward trend in recent years due to developments in medical technology and evolving lifestyle patterns.^4^ This positive trend has led to a steady increase in the number of cancer survivor which be defined as an individual from the diagnosis and throughout life.^5^ With the increase of survival rates, the care needs of cancer survivors have gradually shifted from increased longevity to improved quality of life and a less uncomfortable experience.^5^ After completed primary treatments, cancer survivors are still suffering from a series of physical symptoms such as pain, fatigue and nausea,^6^, ^7^, ^8^ and psychological symptoms including anxiety, depression and irritability,^9^, ^10^, ^11^ due to the influence of treatment modalities as well as the disease itself.^12^ It was shown that cancer survivors may experience six to nine physical and mental symptoms simultaneously and cancer survivors face a significant burden of disease management due to the persistence and interplay of these symptoms.^13^ Consequently, the management of these multifaceted issues has emerged as a critical focus in contemporary cancer survivorship care. Despite the growing recognition of cancer survivorship issues, such as symptom management, quality of life improvement, the systematic development of comprehensive programs to assist healthcare professionals in managing cancer survivors remains limited. To address this gap and facilitate the creation of effective service systems that mitigate the impact of cancer on surviving patients’ lives, Fitch et al. proposed a supportive care framework and defined supportive care needs (SCNs) as the multidimensional assistance required to address complications and side effects throughout the entire disease trajectory.^14^ Subsequent research has extensively explored the SCNs of cancer survivors, with accumulating evidence demonstrating that survivorship care programs tailored to these specific needs can optimize resource utilization and significantly alleviate the care burden experienced by cancer survivors.^15^^,^^16^

At present, most studies investigated the SCNs at one point in time and analyzed the influencing factors,^17^^,^^18^ with a view to clarifying patients' SCNs and thereby improving their quality of life. However, according to supportive care theory, patients’ SCNs exhibit dynamic changes with varying emphasis across different disease stages.^14^ In response to this theoretical perspective, an increasing number of researchers have recently shifted their focus to longitudinal investigations of SCNs, employing diverse statistical methodologies to identify change patterns. Conventional approaches, such as repeated measures analysis of variance,^19^ hierarchical modeling,^20^ and latent growth curve modeling (LGCM),^21^ based on the assumption of population homogeneity, but it is difficult to fulfill in reality. However, recent advancements in statistical methodologies have led to the development of innovative approaches for analyzing longitudinal data.

Group-based trajectory modeling, which is a relatively new way to addresses the limitations inherent in traditional statistical methods. Unlike conventional approaches, these methods operate under the fundamental assumption of population heterogeneity, thereby enabling the identification of distinct subgroups characterized by similar trajectory patterns.^22^ The advent of these methods has undoubtedly provided an effective tool for analyzing differences in individual development trends and gain deeper insights into the unique growth characteristics of various subgroups.^23^ Additionally, the group-based trajectory modelling approach can handle nonlinear and complex trajectory fitting more flexibly than traditional methods, which means it allows each latent class to have an independent trajectory form, whereas traditional methods require a fixed functional form (e.g., linear, quadratic) to be pre-specified.^24^ Most importantly, the group-based trajectory modelling approach can visualize the subgroup trajectory maps instead of using abstract parameters to express the trend as in the traditional approach. The application of group-based trajectory modeling in medical research has gained significant traction, with an increasing number of studies utilizing this methodology to investigate SCNs trajectories among cancer survivors.^25^^,^^26^

However, there are differences in the trends in SCNs of cancer survivors due to the study design (statistical methods, duration of follow-up) of each study, as well as the heterogeneity of the population due to different demographic and sociological backgrounds and differentiated disease backgrounds (cancer type, stage). Notably, we can see that there exists a significant gap in research that comprehensively synthesizes both the trajectories of SCNs and their influencing factors among cancer survivors. Moreover, the growing body of evidence demonstrating substantial cross-cultural and regional variations in SCNs,^27^ especially sexuality needs, which was a more private subject for Asian populations, and therefore the findings will generally be lower than the actual situation.^28^ China, as the world's most populous nation, accounts for approximately 20% of the world's cancer survivor population indicating that more attention should be attached to the SCNs of Chinese cancer survivors.^29^ Due to the lagging nature of clinical practice and individualized care, a lack of comprehensive understanding of the changing trends in the SCNs of Chinese cancer survivors may lead to clinical neglect of the critical needs of specific groups at specific stages or even lead to a misallocation of care resources.

Therefore, to address the cultural and regional variability in SCNs and provide more targeted insights, this review will specifically concentrate on Chinese cancer survivors. We will systematically examine studies that have employed group-based trajectory modeling approaches to investigate SCNs patterns in this population. Furthermore, this review aims to synthesize evidence regarding the key factors influencing the development of distinct trajectory categories, thereby providing healthcare professionals with valuable insights for designing culturally appropriate prevention and intervention strategies.

## Methods

This review was registered with the International Prospective Register of Systematic Reviews on 3 September 2024 (Registration No. CRD42024586557) and was reported following the PRISMA reporting checklist reporting.^30^

### Searching strategy

The electronic databases including CINAHL, Cochrane Library, Embase, PubMed, Web of Science, Scopus, CNKI, Sinomed, Vip Database, Wanfang database were systematically searched from databases inception to 16 October 2024. A combination of search terms “supportive care needs/supportive care/unmet needs/needs assessment/psychosocial care needs/social needs/emotional needs/physical needs/work needs/employment needs”, “trajectory/longitudinal studies/cohort studies/prospective studies”, “cancer/neoplasms/tumor” were used. The detailed search strategy was shown in the supplementary materials (Appendix A).

### Eligibility and exclusion criteria

The inclusion criteria included: (1) longitudinal studies using group-based trajectory modeling approaches such as latent growth mixed model (LGMM), and latent class growth analysis (LCGA)/latent class growth model (LCGM); (2) the population were adult cancer survivors ≥ 18 years old; (3) the outcome indicator was the SCNs trajectory of cancer patients measured by the validated scale; (4) the number of assessment points ≥ 3. Studies were excluded if they (1) were the type of articles such as expert commentary and opinions, conference abstract, or protocols; (2) were not obtained the full text; (3) published in languages other than Chinese and English.

### Study selection

Literatures were screened independently by two researchers based on the title and abstract. After reading the title and abstract to exclude irrelevant literatures, the full text was further read to determine whether to include the literature. Any disagreements were discussed with a third researcher. EndNote X9.1 software was used to screen studies.

### Data extraction and assessment of study quality

Two reviewers extracted data from the eligible studies by using a standardized data collection form. Data extraction information included the first author, year of publication, country/region of study, study design, sample, assessment tool, validation of the assessment tool, the number and timing of assessments, length of follow-up, number and pattern of trajectories identified, risk factors associated with the trajectories, the statistical approach used, and how missing data was dealt with. Studies that met the eligibility criteria were evaluated by two tools: The JBI critical appraisal tools for cohort studies^31^ and GRoLTS (Guidelines for Reporting on Latent Trajectory Studies).^32^ The JBI critical appraisal checklist for cohort studies has 11 items, each of which was evaluated according to ‘yes’, ‘no’, ‘unclear’, and ‘not applicable’. Then, we calculated the proportion of each study's total ‘yes’ to the total item for the included literature. Specific elements of the criteria for evaluating the tool are provided in the supplementary material (Appendix B). The included literature was evaluated as high quality (the percentage of ‘yes’ greater than 70%), moderate (the percentage of ‘yes’ between 50% and 70%) or low (the percentage of ‘yes’ less than 50%). In addition, GRoLTS checklist was used to evaluate the quality of the process of latent trajectory analysis. The GRoLTS checklist included 16 items (some with subitems) and each item was scored 0 (not reported) or 1 (reported). The studies scored ≤ 10 were considered as low quality, 11–16 were moderate quality and 17–21 were high quality. The quality of the literature was evaluated independently by two researchers. Any disagreement would be discussed with the third researcher until reach a consensus.

### Synthesis of data

Narrative synthesis methods instead of meta-analysis were used to integrate the outcomes of articles due to significant differences in participants, assessment points, methods of trajectory analysis and so on. In this review, we have predefined five trajectory categories. The first category, identified as the “high-stable trajectory”, was defined as persistently elevated SCNs levels than other trajectory categories with no significant change over time. The second category, termed “moderate-stable trajectory”, was had an intermediate SCNs level consistently compared with survivors in other categories. The third trajectory pattern, designated as “low-stable trajectory”, demonstrated consistently low SCNs levels than other categories. The fourth category, which survivors experienced a gradual decrease in SCNs over time, was named as “decreasing trajectory”. Finally, the fifth trajectory was characterized by survivors reporting progressively higher SCNs over time and was labeled “increasing trajectory”. Meanwhile, considering the diversity of the factors influencing SCNs trajectories explored in the included studies, SEM (social-ecological model) was used to summarize these factors in this review.

## Results

**Fig. 1:**
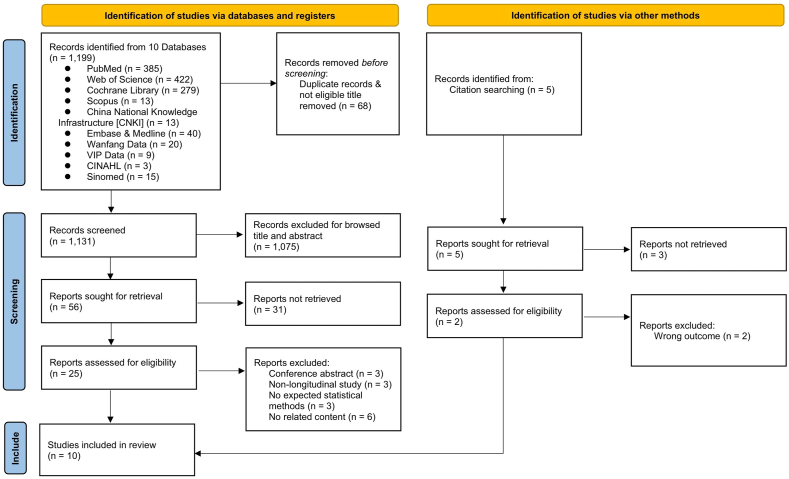
PRISMA flow diagram for study selection. PRISMA, Preferred Reporting Items for Systematic Reviews and Meta-Analyses.

A total of 1204 initial records were identified by searching through the literature search, including 1199 from the nine databases and five from citation searching. After excluding duplicates and those which were not related to the theme after reading the article titles and abstracts, 25 records were eligible for screening the full text. After reviewing the full texts, 15 studies were excluded mainly because of ineligible genre (*n* ​= ​3), ineligible study design (*n* ​= ​3), ineligible statistical method (*n* ​= ​3), unrelated content (*n* ​= ​6). Ultimately, 10 records for narrative synthesis. The study selection process is presented in Fig. 1.

### Details of included studies

All the studies included in our study were published between 2022 and 2024, and they were all conducted in China.^33^, ^34^, ^35^, ^36^, ^37^, ^38^, ^39^, ^40^, ^41^, ^42^ There was total 1842 participants included in the 10 studies, ranging from 71 to 249 per study. In the included studies, 3 studies focused on lung cancer patients,^34^^,^^37^^,^^42^ 2 studies concentrated on colorectal cancer patients,^39^^,^^41^ and 2 studies focused on bladder cancer patients,^35^^,^^38^ respectively. All the included studies were prospective. Table 1 shows the characteristics of the included studies. The Supportive Care Needs Survey Short Form 34 (SCNS-SF34) was the most commonly used tool for assessing SCNs, and the Short Form Survivor Unmet Needs Survey (SF-SUNS) was used by two studies.^34^^,^^40^ In addition, only one study used the Needs Evaluation Questionnaire–Chinese version (NEQ-C) to evaluate SCNs in cancer survivors.^42^ The majority of studies investigated changes in patients’ SCNs before and after surgery, with only three studies investigating SCNs during postoperative adjuvant therapy.^34^^,^^40^^,^^42^ Additionally, the number of assessments was between 3 and 8 with a median of 4 and the duration of follow-up ranged from 3 months to 9 months. Five studies clearly reported the basis for the selection of follow-up time points,^33^, ^34^, ^35^^,^^41^^,^^42^ mainly based on clinical treatment or expert consensus to determine the interval of follow-up, while others did not explain the reasons for the follow-up time settings.

**Table 1:** The characteristics of the included studies.

Author, time, country	Study design	Study population	Sample sources	Sample size (used for trajectory analysis)	Assessment tool	Validation of the assessment tool	The number and timing of assessments	Length of follow-up
Qin, 2022, China^33^	Prospective cohort study	Esophageal cancer patients	The department of thoracic surgery of the first affiliated Hospital of Soochow University	215	SCNS-SF34	Cronbach’α ranged from 0.874 to 0.951	4 points: Pre-operation (T0) 1–2 days before discharge (T1) 1 month after discharge (T2) 3 months post-operation (T3)	3 months
Wang, et al., 2022, China^34^	Prospective cohort study	Lung cancer patients	The oncology wards of 4 tertiary hospitals in Harbin, Xi'an and Beijing	226	SF-SUNS	Cronbach's α ​= ​0.894	3 points: 1 day before the first chemotherapy (T1) 1 day before the third chemotherapy (T2) after the chemotherapy to re-examination (T3)	9 months
Xiao, 2022, China^35^	Prospective cohort study	Bladder cancer patients	The department of Urology and oncology of three tertiary hospitals in Henan province	156	SCNS-SF34	Cronbach's α ​= ​0.947	4 points: 1–2 days after surgery (T1) 2 month after surgery (T2) 6 month after surgery (T3) 8 months surgery (T4)	8 months
Wang, et al., 2023, China^36^	Prospective cohort study	Elderly breast cancer patients	Hospitals in Shanghai	122	SCNS-SF34	Cronbach's α ​= ​0.92	4 points: the period before surgery (T1) 14 days after surgery (T2) 3 months after surgery (T3) 6 months after surgery (T4)	6 months
Liu et al., 2023, China^37^	Prospective cohort study	Non-small cell lung cancer patients	Shanghai Lung Hospital affiliated to tongji University	177	SCNS-SF34	Cronbach's α ​= ​0.84	5 points: 1 day before operation (T0) 3 days after operation (T1) 1 day before discharge (T2) 1 week after discharge (T3) 1 month after discharge (T4) 3 months after discharge (T5)	3 months
Yan et al., 2024, China^38^	Prospective cohort study	Patients with urinary stoma of bladder cancer	Department of Hepatobiliary Urology, Hainan Hospital of Chinese PLA general Hospital	174	SCNS-SF34	Cronbach's α ​= ​0.86	4 points: the day of discharge (T1) 1 month after ostomy (T2) 3 months after ostomy (T3) 6 months after ostomy (T4)	6 months
Zhang et al., 2024, China^39^	Prospective cohort study	Low-lying rectal cancer patients	Department of gastrointestinal surgery, the affiliated Hospital of Qingdao University	249	SCNS-SF34	Cronbach's α ​= ​0.89	3 points: 1 month after surgery (T1) 3 months after surgery (T2) 6 months after surgery (T3)	6 months
Zhu et al., 2024, China^40^	Prospective cohort study	Patients with head and neck malignancies	Department of radiotherapy, the first affiliated Hospital of School of medicine, Xi'an Jiaotong University	220	SF-SUNS	Cronbach's α ​= ​0.894	4 points: before radiotherapy (T1) After radiotherapy (T2) 3 months after radiotherapy (T3) 6 months after radiotherapy (T4)	6 months
Zhou et al., 2024, China^41^	Prospective cohort study	Patients undergoing colorectal cancer surgery with enterostomy	three tertiary care hospitals (Peking University People's Hospital, Peking University cancer Hospital and China–Japan Friendship Hospital) in China	232	SCNS-SF34	Cronbach's α ​= ​0.89	4 points: before discharge (T1) The first week of discharge (T2) The first month of discharge (T3) The third month of discharge (T4)	3 months
Yen et al., 2023, China, taiwan^42^	Prospective cohort study	Advanced non-small-cell lung cancer patients	Medical center in southern taiwan	71	NEQ-C	Cronbach's α ​= ​0.92	8 points: 1 day before the first cycles of chemotherapy (T1) 7 day after the end of the first cycles of chemotherapy (T2) 1 day before the second cycles of chemotherapy (T3) 7 day after the end of the second cycles of chemotherapy (T4) 1 day before the third cycles of chemotherapy (T5) 7 day after the end of the third cycles of chemotherapy (T6) 1 day before the fourth cycles of chemotherapy (T7) 7 day after the end of the fourth cycles of chemotherapy (T8)	4 months

SCNS-SF34, The Supportive Care Needs Survey Short Form 34; SF-SUNS, Short Form Survivor Unmet Needs Survey; NEQ-C, Needs Evaluation Questionnaire–Chinese version.

### Study quality

**Fig. 2:**
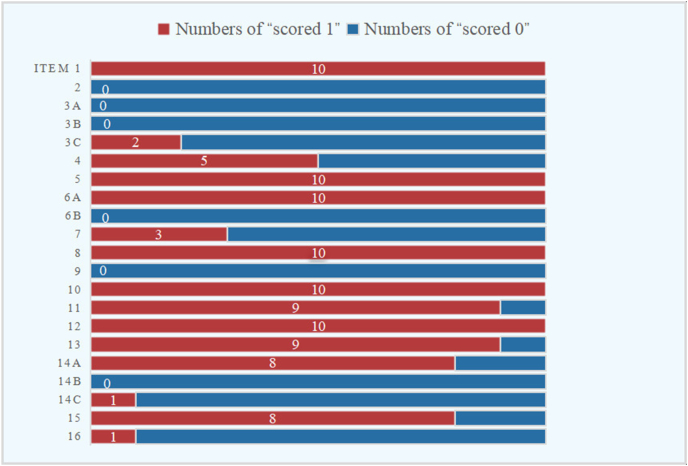
The results of the GRoLTS checklist. GRoLTS, Guidelines for Reporting on Latent Trajectory Studies.

According to the JBI critical appraisal tools for cohort studies, 2 studies were considered high quality,^33^^,^^36^ and only 2 studies were deemed of low quality.^34^^,^^42^ The complete results of the evaluation of the JBI critical appraisal tools for cohort studies are shown in Supplementary Materials (Appendix C). Moreover, the results and the details of the evaluation of the GRoLTS checklist were illustrated in Supplementary Materials (Appendix D). 4 studies were considered as low quality,^34^^,^^37^^,^^40^^,^^42^ and other studies were all evaluated as moderate quality.^33^^,^^35^^,^^36^^,^^38^^,^^39^^,^^41^ Of all the 21 items, those with a reporting rate of more than 50% include metric of time used in the statistical model reported (item 1), the distribution of the observed variables (item 4), the software used (item 5), the methods of dealing with within-class heterogeneity (item 6a), how covariates were included (item 8), statistical perspective used in model comparison (item 10), the total number of fitted models (item 11), the number of cases per class reported for each model (item 12), entropy reported (item 13), the plot of the estimated mean trajectories of the final solution (item 14a), and whether characteristic of the class solution was described numerically (item 15). But none of the following items were reported: the mean and variance of time within a wave (item 2), the missing data mechanism (item 3a), the description of variables related to attrition/missing data (item 3b), the between-class differences in variance-covariance matrix structure (item 6b), the number of random start values and final iterations (item 9), and the estimated mean trajectories for each model (item 14b). Of the 10 articles included in this study, six of the items in the GRoLTS checklist had the highest rate of reporting: the metric of time used in the statistical model (item 1), software used (item 5), within-class heterogeneity (item 6a), how covariates were included (item 8), and model comparison tools described from a statistical perspective (item 10), numbers of cases for per class (item 12) (Fig. 2). In general, most of the included studies were evaluated as medium quality. The highest score was 13 and the average score of 10 articles was 10.60.

### SCNs trajectories in the included studies

#### Identification and screening of the SCNs trajectories

The statistical methods used by studies conducting group-based the SCNs trajectory analysis are shown in Supplementary Materials (Appendix E). Latent class growth model (LCGM)/latent class growth analysis (LCGA) was the most commonly used trajectory modeling method (*n* ​= ​7), and two studies used the latent growth mixed model (LGMM)/growth mixed model (GMM). In addition, the study by Yen used group-based trajectory modeling (GBTM) to identify the classes of SCNs trajectories.^42^ Akaike Information Criterion (AIC), Bayesian Information Criterion (BIC), adjusted Bayesian Information Criterion (aBIC), Lo-Mendell-Rubin likelihood ratio test (LMR-LRT) and Bootstrap likelihood ratio Test (BLRT) were reported in almost every study for model selection. In addition, Sample-Size Adjusted Bayesian Information Criteria (SABIC), Sample-Size Bayesian Information Criteria (SSBIC), sample size; clinical interpretability and model convergence were infrequently used relatively. Furthermore, most included studies were not reported how to deal with missing value, but there were only two studies reported to use the methods of holographic great likelihood estimation,^36^ and eliminating those with more than 20% omissions to solve the missing data.^33^

#### Characterization of the SCNs trajectories

The characteristics and distribution of SCNs trajectories are summarized in Table 2. The review findings indicate that the majority of cancer survivors exhibited a general decline in SCNs following surgical intervention, while SCNs levels remained persistently elevated during the postoperative adjuvant therapy phase. Analysis of the included studies revealed that most articles identified three distinct trajectories, and the naming rules were varied. Nine studies adopted a naming convention based on the relationship between baseline SCNs scores and trajectory slopes,^33^, ^34^, ^35^, ^36^, ^37^, ^38^^,^^40^, ^41^, ^42^ while one study exclusively utilized slope characteristics without reference to baseline scores.^39^ Notably, two studies incorporated both the direction (positive/negative) and magnitude of slope values in their trajectory classification.^38^^,^^39^ Of particular methodological significance is the observation that only two studies employed standardized SCNs assessment tool cutoffs to categorize trajectories as high/medium/low,^34^^,^^40^ with the remaining studies relying on relative comparisons between trajectories for classification purposes.

**Table 2:** Summary of group-based SCNs trajectory and its influencing factors.

Studies	Pattern and class of trajectories	Methods of identifying predictors	Statistically significant factors				
			Demographic variables	Clinical variables	Personal symptoms	Social support	Healthcare provision and community level factors
Qin, 2022^33^	Overall, with 4 trajectories: high-level stability group (18.8%) High-level decline group (40.2%) Low-level rise (20.9%) Low-level stability group (20.0%)	Univariate analyses; Multiple logistic regression	1. Age 2. Monthly household income	1. Treatment methods 2. Nutritional status 3. Length of diagnosis 4. Comorbidities	1. Baseline depression 2. Baseline anxiety	1. Baseline family care	1. Place of residence
Wang, et al., 2022^34^	Overall, with 5 trajectories: the high-needs rise group (11.5%) The medium-needs stable group (32.3%) The high-needs stable group (43.7%) The low-needs rise group (6.6%) The high-needs decrease group (5.8%)	Analysis of variance, ANOVA/The chi-square test	1. Age 2. Educational level	NR	1. Baseline depression	1. Baseline family care	NR
Xiao, 2022^35^	Overall, with 2 trajectories: 1. low demand moderate decline group (56%) 2. High demand declined group (44%)	Univariate analyses; multivariate binary logistic regression analyses	1. Age 2. Educational level	NR	1. Baseline depression	1. Baseline family care	NR
Wang, et al., 2023^36^	Overall, with 3 trajectories: high needs decline group (38.5%) High needs sustained group (51.6%) low needs sustained group (9.8%)	Univariate analyses; Multi-categorical logistic regression analysis	NR	1. Treatment protocols	1. Personality traits	1. Primary caregiver	NR
Liu et al., 2023^37^	Overall, with 3 trajectories: high demand group (46.33%) The slowly decreasing demand group (30.51%) The low decreasing demand group (23.16%)	The chi-square test; multi-categorical logistic regression analysis	1. Education level	1. Comorbidities 2. Disease stage	1. Psychological distress	1. Social support	NR
Yan et al., 2024^38^	Overall, with 3 trajectories: high-demand slow-decline group (43%) Medium-demand rapid-decline group (33%) Low-demand slow-rise group (24%)	The chi-square test/Kruskal–Wallis test; multi-categorical logistic regression analysis	1. Age 2. Education level	1. Comorbidities	1. Fear of disease of progression	1. Social support	NR
Zhang et al., 2024^39^	Overall, with 3 trajectories: the persistently high group (20.5%) The rapidly descending group (43.8%) The slowly descending group (35.7%)	Analysis of variance, ANOVA/The chi-square test; multi-categorical logistic regression analysis	1. age 2. Education level 3. Monthly household income	1. Treatment methods 2. Complications 3. Comorbidity	1. Psychosocial adjustment	NR	NR
Zhu et al., 2024^40^	Overall, with 3 trajectories: the high demand reduction group (31.36%) The high-demand stable group (35%) The low-demand rising group (33.64%)	The chi-square test; multi-categorical logistic regression analysis	1. Age 2. Years of education 3. Monthly household income	NR	NR	1. Social support	NR
Zhou et al., 2024^41^	Overall, with 3 trajectories: low needs decline group (57.3%) High needs slow decline group (19.4%) Needs rapid decline group (23.4%)	Univariate analyses; multinomial logistic regression	1. Educational level	1. Type of surgery	1. Psychosocial adjustment	NR	NR
Yen et al., 2023^42^	Overall, with 3 trajectories: low-unstable group (19.7%) Moderate-stable group (43.7%) High-stable group (36.6%)	The chi-square test	1. Marital status	NR	NR	NR	NR

SCNs, supportive care needs; NR, not report.

The comprehensive analysis of 10 studies revealed five distinct SCNs trajectory patterns. The first category, identified as the “high-stable trajectory” (*k* ​= ​7),^33^^,^^34^^,^^36^^,^^37^^,^^39^^,^^40^^,^^42^ was characterized by persistently elevated SCNs levels with minimal temporal variation. The proportion of samples in this trajectory category ranged from 18.8% to 51.6%, with a total prevalence of 23.78% after recategorization. The second category, termed “moderate-stable trajectory” (*k* ​= ​2), was exclusively observed in lung cancer populations,^34^^,^^42^ with sample proportions of 32.3% and 43.7% respectively, yielding a combined prevalence of 5.65%. The third trajectory pattern, designated as “low-stable trajectory” (*k* ​= ​2),^33^^,^^36^ demonstrated consistently low SCNs levels, accounting for 9.8%–20% of study samples and an overall prevalence of 2.99%. Nine studies identified decreasing SCNs patterns, collectively referred to as “decreasing trajectory” (*k* ​= ​16),^33^, ^34^, ^35^, ^36^, ^37^, ^38^, ^39^, ^40^, ^41^ which encompassed various initial levels (high, moderate, low) and rates of decline (moderate, slow, rapid).^35^^,^^37^, ^38^, ^39^^,^^41^ Notably, while Yen et al. reported a trajectory with initial increase followed by fluctuation,^42^ its terminal SCNs level remained below baseline, warranting its classification within the decreasing trajectory category. The percentage of participants in the decreasing trajectory ranged from 5.8% to 56%, with a total prevalence of 58.90% after recategorization; The fifth trajectory pattern, identified in four studies,^33^^,^^34^^,^^38^^,^^40^ was characterized by baseline SCNs levels (either high or low) followed by an upward trend, thus termed “increasing trajectory” (*k* ​= ​5). This category comprised 6.6%–33.64% of study samples, with a combined prevalence of 8.69%.

### Factors influencing the trajectory heterogeneity of SCNs

Among the reviewed studies, ten investigations examined factors influencing SCNs trajectories, with the majority employing a combination of univariate analyses and multivariate logistic regression as their primary analytical approach. While two studies relied exclusively on univariate analyses to identify determinants of trajectory categories,^34^^,^^42^ these were excluded from the current review to ensure robust control for potential confounding variables.

SEM provides a comprehensive theoretical framework for understanding the multi-level influence of socio-environmental factors on human behavior,^43^ and has gained significant traction in cancer research. McIntosh's seminal work on prostate cancer patients' SCNs demonstrated the utility of SEM in analyzing SCNs determinants through the lens of multi-level interactions spanning individual, social, and environmental dimensions.^44^ Given the multifaceted nature of SCNs development and progression, this review adopts the SEM framework to systematically categorize and present the identified influencing factors of SCNs trajectories, as detailed in Table 2.^45^

#### Demographic characteristics

Demographic factors, particularly age, education level, and monthly household income, emerged as significant predictors of SCNs trajectories across multiple studies. The relationship between age and SCNs patterns demonstrated complexity, with most studies indicating that older patients were more likely to exhibit increasing or high-stable SCNs trajectories (OR ​= ​1.262, 95% CI: 1.040–1.531; OR ​= ​5.764, 95% CI: 1.854–17.915; OR ​= ​1.076, 95% CI: 1.008–1.149; OR ​= ​0.748, 95% CI: 0.671–0.835).^33^^,^^38^, ^39^, ^40^ However, Xiao's study presented a contrasting finding, suggesting that older patients might follow a high-demand decline trajectory (OR ​= ​0.960, 95% CI: 0.93–0.99).^35^ Educational level consistently demonstrated predictive value, with six studies reporting statistically significant results that converged on a consistent pattern: patients with higher education levels were more likely to experience downward trends in SCNs (OR ​= ​0.160, 95% CI: 0.04–0.60; OR ​= ​6.076, 95% CI: 1.419–26.011; OR ​= ​6.844, 95% CI: 1.225–38.253; OR ​= ​0.184, 95% CI: 0.048–0.710; OR ​= ​1.619, 95% CI: 1.220–2.150; OR ​= ​0.325, 95% CI: 0.115–0.920).^35^^,^^37^, ^38^, ^39^, ^40^, ^41^ The influence of monthly household income on SCNs trajectories was examined in eight studies, with three reporting statistically significant associations.^33^^,^^39^^,^^40^ These studies collectively identified lower household income as a risk factor, typically associated with either persistently high SCNs levels (OR ​= ​0.059, 95% CI: 0.016–0.212)^33^ or increasing SCNs over time (OR ​= ​4.023, 95% CI: 1.340–12.072).^40^ While Zhang's study observed a decreasing pattern among low-income patients, the decline occurred at a notably slower rate compared to other groups.^39^ Notably, several demographic factors failed to demonstrate statistically significant effects on SCNs trajectories in the reviewed studies, including marital status, gender, smoking and drinking habits, body mass index (BMI), and family history of malignant neoplasms.

#### Clinical variables

Clinical characteristics and treatment-related factors significantly influenced SCNs trajectories across multiple studies in this review, including treatment modalities, comorbidities, clinical stage, complications, nutritional status, time since diagnosis, and surgical type.^33^^,^^35^, ^36^, ^37^, ^38^, ^39^^,^^41^^,^^46^ Treatment methods emerged as a particularly influential factor, with three out of five studies examining this aspect reporting statistically significant associations.^33^^,^^36^^,^^39^ These studies consistently demonstrated a positive correlation between treatment complexity and SCNs levels. Specifically, Qin's study revealed that patients undergoing combined treatment modalities (surgery, chemotherapy, and radiotherapy) were more likely to maintain persistently high SCNs levels (OR: 17.734, 95% CI: 1.211–259.709).^33^ This finding was corroborated by Wang's research, which identified elevated SCNs among patients receiving multiple treatment modalities including surgery, chemotherapy, hormonal therapy, and radiotherapy (OR: 161.246, 95% CI: 5.085–5112.917).^36^ Furthermore, Zhang's study highlighted that patient receiving adjuvant chemotherapy following surgery exhibited either prolonged high SCNs levels or slower decline rates compared to those undergoing surgery alone (OR: 2.792, 95% CI: 1.143–6.817).^39^ The impact of comorbidities on SCNs trajectories was consistently observed, with most studies indicating that patients with multiple comorbidities tended to maintain higher SCNs levels or experience slower declines over time (OR: 2.850; 95% CI: 1.076–7.546; OR: 5.148; 95% CI: 1.715–15.456; OR: 0.293; 95% CI: 0.110–0.781).^37^, ^38^, ^39^ Qin's study further suggested a progressive increase in SCNs among patients with greater comorbidity burden (OR: 4.564; 95% CI: 1.051–18.800).^33^ Additional clinical factors influencing SCNs patterns included nutritional status and disease stage, with malnourished patients and those with stage III cancer demonstrating consistently elevated SCNs levels (OR: 0.277, 95% CI: 0.161–0.476; OR: 18.526, 95% CI: 3.633–94.472).^33^^,^^37^ In addition, it was reported that the SCNs of patients with postoperative complications may show a descend trend but slowly (OR: 0.226, 95% CI: 0.060–0.843)^39^ and those who with shorter cancer diagnoses show a high level SCNs but with a decreasing trend (OR: 10.236, 95% CI: 2.279–45.982).^33^ Notably, Zhou's study identified surgical type as a significant factor, with patients undergoing temporary enterostomy showing more favorable SCNs trajectories over time.^39^

#### Personal symptoms

In the aspects of personal symptoms, four factors were emerged as significant determinants of SCNs trajectories, including depression, anxiety, fear of disease of progression and psychosocial adjustment ability. The high-stable SCNs trajectory was consistently associated with elevated levels of depression (OR: 4.830, 95% CI: 2.296–10.161; OR: 1.620, 95% CI: 1.25–2.11; OR: 0.014, 95% CI: 0.002–0.114),^33^^,^^35^^,^^36^ and anxiety (OR: 4.521, 95% CI: 1.625–12.582).^33^ Furthermore, patients exhibiting greater fear of disease progression were more likely to demonstrate slowly decreasing SCNs patterns (OR: 1.234, 95% CI: 1.128–1.350).^38^ Notably, psychosocial adjustment capacity demonstrated a protective effect, as evidenced by two studies showing that patients with superior psychosocial adaptation skills tended to experience lower SCNs levels over time (OR: 1.113, 95% CI: 1.017–1.129; OR: 1.102, 95% CI: 1.045–1.162).^39^^,^^41^

#### Social support

The influence of social support on SCNs trajectories was examined through multiple studies, revealing complex and sometimes contrasting patterns. Three investigations utilizing the Social Support Rating Scale (SSRS) demonstrated varying effects of social support on SCNs development. Liu's study identified an association between low social support and the “high demand stable group” trajectory (OR: 0.826, 95% CI: 0.737–0.927),^37^ and Yan's research corroborated this finding by showing that higher social support correlated with decreased SCNs over time (OR: 1.143, 95% CI: 0.733–0.915).^38^ However, Zhu's study presented a contrasting result, suggesting that patients with robust social support were more likely to follow a rising needs trajectory (OR: 1.450, 95% CI: 1.283–1.638).^40^ Furthermore, three additional studies explored specific aspects of caregiving and their impact on SCNs patterns. These studies consistently demonstrated that superior family care was associated with either consistently low or progressively decreasing SCNs levels (OR: 0.269, 95% CI: 0.100–0.723; OR: 0.78, 95% CI: 0.63–0.97).^33^^,^^35^ Notably, the caregiver's identity emerged as a significant factor, with patients who served as their own primary caregivers exhibiting persistently higher SCNs compared to those receiving care from spouses or children (OR: 2.894, 95% CI: 0.479–17.489).^36^

#### Healthcare provision and community level factors

At the healthcare system and community levels, two primary factors were investigated: place of residence and health insurance type. However, only the factor of residence location demonstrated statistically significant associations with SCNs trajectories. Specifically, the analysis revealed that patients residing in rural areas were more likely to follow a high-level decline trajectory rather than a low-level stability pattern (OR: 3.528, 95% CI: 1.083–11.490).^33^

## Discussion

This study represents the first systematic review to comprehensively examine SCNs trajectories in cancer survivors utilizing group-based trajectory modeling, while also synthesizing influencing factors through the SEM framework. Our analysis encompassed 10 studies involving 1842 participants across various cancer types, leading to the identification and reclassification of SCNs into five distinct trajectory categories. However, significant heterogeneity was observed in the outcomes, attributable to variations in statistical methodologies, measurement instruments, and study durations. Furthermore, the methodological quality assessment revealed that most included studies demonstrated moderate quality, necessitating cautious interpretation of the findings.

### Main findings of SCNs trajectories

Previous systematic reviews have established that cancer survivors' SCNs exhibit significant variation across different treatment phases,^47^ however, the specific patterns of change within each stage remained unclear. In this review, we found that most cancer survivors' SCNs diminished over the postoperative months. This finding is in line with previous studies, which noted that most cancer survivors reported higher SCNs early after treatment.^48^ Survivors gradually return to normal social life after surgery may contribute to this outcome. Furthermore, our analysis revealed that SCNs remain persistently high during the postoperative adjuvant therapy phase. This sustained elevation may be explained by the prolonged impact of treatment-related side effects on survivors’ functional capacity, occupational performance, and social role fulfillment.^49^ These findings underscore the critical importance of prioritizing actively treated cancer survivors in healthcare service delivery to optimize quality of life outcomes.

Furthermore, this systematic review revealed that the identified SCNs trajectories were categorized into 2 to 4 distinct groups, with the majority of studies converging on a three-group classification. The short follow-up period as well as the small sample size may be the reason for only a small number of trajectory groups being established.^50^^,^^51^ Additionally, the establishment of follow-up intervals in longitudinal studies is an important component of study design and also has a clear impact on the shape and number of trajectories.^32^ However, our study found that only some of the studies explicitly reported the basis for the selection of follow-up time points, which reveals a shortcoming of the existing studies and may explain the heterogeneity of the results across studies. Our analysis also highlighted that most of the included studies used LCGM/LCGA as a statistical method, while only two studies used GMM as a statistical method. LCGM/LCGA was assumed that individuals in the heterogeneous groups have identical growth trajectories, whereas GMM allows for variability among individuals in different categories.^52^ Thus, the use of statistical methods may also have contributed to the low number of trajectory groups. Despite substantial heterogeneity across studies, we successfully synthesized the trajectory findings into five principal categories based on their characteristic patterns: high-stable, moderate-stable, low-stable, decreasing, and increasing trajectories. The prevalence of these five categories was recategorized and we found that the decreasing trajectory reported the greatest share. This finding aligns with previous research utilizing conventional methods to estimate average SCNs trajectories,^53^ suggesting that a substantial proportion of cancer survivors demonstrate the capacity to effectively manage disease-related needs over time. However, this review was unable to elucidate potential heterogeneity in the rate of SCNs reduction among survivors, indicating an important area for future research.

With the development of methods for analyzing longitudinal data, latent trajectory analysis is becoming more and more popular to analyze longitudinal data. To enhance the consistency of studies employing latent trajectory analysis and to facilitate comparisons and replications across studies — thereby enabling systematic reviews and meta-analyses of similar research — the GRoLTS was developed.^32^ Reporting standards are pivotal to research quality, as evidenced by a systematic review concluding that their use significantly enhances the rigor of studies.^54^ In latent trajectory studies, the extent of GRoLTS reporting directly correlates with the reliability of the outcomes. However, the mean GRoLTS checklist score among the included studies was only 10.60, reflecting moderate quality. The factors contributing to this mainly included the absence of reporting the mean and variance of time within a wave, the uncertainty of missing data mechanism, no description of variables related to missing data, fail to considerate and document the across-class differences in variance–covariance matrix clearly, unable to report the number of random start values and plots for each model. GRoLTS checklist, which was an important protocol to guide the research of using latent trajectory analysis, was released in 2016. Unfortunately, although all the included studies were published after 2016, the shortcomings in methodology and reporting ways still remain. Consequently, it is recommended to design and report in accordance with the item of GRoLTS checklist to maximize the validity and reproducibility of articles.

### Main findings of the factors of SCNs trajectories

#### Demographic variables

Regarding demographic variables influencing trajectory group classification, our analysis identified nine potential factors across included studies: age, education level, monthly household income, gender, occupation, smoking/drinking habits, marital status, activities of daily living, and family history of malignant neoplasms. Notably, only age, education level, and monthly household income demonstrated statistically significant associations.

##### Age

The role of age in shaping SCNs trajectories emerged as particularly complex. While half of the ten studies identified age as statistically significant, its directional impact remains contested. Most investigations suggested younger patients predominantly clustered in “declining trajectory groups",^33^ whereas older adults tended to maintain elevated SCN levels or demonstrate upward trajectories over time (OR ​= ​1.262, 95% CI: 1.040–1.531; OR ​= ​5.764, 95% CI: 1.854–17.915; OR ​= ​1.076, 95% CI: 1.008–1.149; OR ​= ​0.748, 95% CI: 0.671–0.835).^38^, ^39^, ^40^ However, the findings of Xiao led to a different conclusion which was considered that the SCNs of older patients decreased over time (OR ​= ​0.960, 95% CI: 0.93–0.99).^35^ To explain this discrepancy, we reviewed related literatures which identified potential mechanisms: older adults may exhibit lower-than-true levels of SCNs due to concerns about family burdens or difficulties in authentically expressing sexual needs due to traditional Chinese culture.^55^ This suggests current measurements may systematically underestimate true SCN prevalence in elderly populations, potentially explaining the observed inconsistencies across studies.

##### Education level

All studies investigated the predictive role of educational attainment on SCNs trajectories, with six demonstrating consensus that higher-educated patients disproportionately clustered in decreasing trajectory groups (OR ​= ​0.160, 95% CI: 0.04–0.60; OR ​= ​6.076, 95% CI: 1.419–26.011; OR ​= ​6.844, 95% CI: 1.225–38.253; OR ​= ​0.184, 95% CI: 0.048–0.710; OR ​= ​1.619, 95% CI: 1.220–2.150; OR ​= ​0.325, 95% CI: 0.115–0.920). Researchers attribute this pattern to education's strong association with health literacy – the capacity to acquire, interpret, and apply essential health information.^56^ Better-educated patients typically exhibit enhanced disease understanding and greater receptivity to medical knowledge, potentially explaining their attenuated SCNs compared to less-educated counterparts. These findings collectively suggest prioritizing health literacy interventions for educationally disadvantaged populations. Common and vivid words should be used to describe disease knowledge to patients with low educational level, avoiding medical jargon as much as possible, and providing need-based care with appropriate content and difficulty.

##### Monthly household income

The analysis revealed a significant association between monthly income levels and SCNs trajectories, with lower-income patients demonstrating greater likelihood of sustained high or progressively increasing SCNs patterns (OR ​= ​0.059, 95% CI: 0.016–0.212; OR ​= ​4.023, 95% CI: 1.340–12.072). This correlation stems from the dual financial burden inherent in cancer management — encompassing both direct treatment costs and indirect expenses such as transportation and income loss^57^ — which disproportionately impacts economically vulnerable populations.^58^ Such financial strain exacerbates both physical and psychological unmet needs through constrained access to care and chronic stress mechanisms. Screening provided by health caregivers, as well as financial assistance, is considered an effective practice to alleviate the financial burden of cancer survivors.^59^

#### Clinical variables

##### Treatment modalities

The results of a previous systematic review showed that different types of treatment create different SCNs, and the SCNs increase from patients having surgery only, surgery and radiotherapy, to surgery, radiotherapy, and chemotherapy.^60^ In this review, we drew similar conclusions from a longitudinal research perspective. The surgical intervention itself represents a significant biopsychosocial stressor,^61^ with subsequent adjuvant therapies introducing compound complications ranging from radiation-induced dermatitis to chemotherapy-associated gastrointestinal sequelae.^62^ Therefore, patients receiving combined therapies more likely tend to face consistently higher or progressively higher SCNs than other patients.

##### Comorbidities

The role of comorbidities in influencing the SCNs trajectory found in this study remains controversial, but the results all suggest that more comorbidities are a risk factor for the SCNs trajectory, which means it will lead to maintain high level of SCNs over time or increasing trend (OR: 2.850; 95% CI: 1.076–7.546; OR: 5.148; 95% CI: 1.715–15.456; OR: 0.293; 95% CI: 0.110–0.781; OR: 4.564; 95% CI: 1.051–18.800). Past research suggested that a diagnosis of multiple comorbidities may adversely affect the healthcare experience of cancer survivors, reporting poorer satisfaction with care.^63^ Additionally, due to the complexity of managing cancer and comorbidities, cancer survivors with comorbidities expressed greater unmet need for supportive care compared to cancer survivors without comorbidities.^64^ The results have similarities with our study that comorbidity factors are risk factors for the trajectory of SCNs. However, systematic and close communication with healthcare providers has been shown to enhance cancer survivors' self-efficacy, largely building cancer survivors’ confidence in overcoming the disease, which in turn helps them address needs.^65^ Based on this, future efforts should be made to promote effective communication between cancer survivors and healthcare providers.

##### Clinical stage

Clinical staging of cancer has been shown to be strongly associated with multifaceted SCNs.^66^ The progression of the cancer disease impairs physical functioning as well as psychological coping, and patients often face a range of problems such as reduced physical functioning, emotional instability, and concentration problems.^67^ As a result, patients with advanced stages of the disease are in greater need of multifaceted supportive care to improve their quality of life. However, our results found that only one study supported the impact of cancer clinical stage on SCNs from a longitudinal perspective.^37^ Therefore, the impact of cancer clinical stage on the trajectory of SCNs should continue to be explored in the future.

##### Others

The length of cancer diagnosis is also thought to significantly influence the trajectory of SCNs, and the shorter the diagnosis the more likely the trajectory of SCNs is to show a high level of gradual decline. To explain this phenomenon, we reviewed literatures and found that Yu's study demonstrated that shortly after a cancer diagnosis, patients generally exhibit high levels of negative self-empathy which in turn increases various aspects of supportive needs.^68^ In traditional Chinese culture, when faced with stressful events, Chinese people tend to favor self-doubt, self-denial and a greater lack of self-compassion, thus leading to higher SCNs in the early stages of cancer diagnosis. To address this circumstance, measures related to self-compassion development, such as positive thinking meditation, should be carried out as soon as possible in the early stages of the disease. In addition, few studies reported nutritional status, complications and type of surgery to significantly influence SCNs trajectory,^33^^,^^39^^,^^41^ suggesting that further studies on these factors should be conducted to identify high-need patients as early as possible.

#### Personal symptoms

Psychological distress has been shown to be positively associated with SCNs in cancer survivors,^69^ and the results of this systematic review by reviewing relevant longitudinal studies showed that depression, anxiety, fear of disease of progression and psychosocial adjustment were the risk factors of SCNs sustain high level in cancer survivors. The protracted treatment cycles and ever-present recurrence risks characteristic of cancer management create sustained psychosocial stressors, compounded by treatment-induced physiological sequelae like chemotherapy-related nausea. This dual burden establishes a self-perpetuating cycle where biopsychosocial stressors intensify emotional distress, which subsequently diminishes psychological resilience and amplifies unmet SCNs through maladaptive coping mechanisms. Given this situation, dynamic and timely assessment of patients’ psychological status was deemed important. Contemporary patient-centered care models emphasize multidisciplinary coordination and continuous therapeutic alliances that prioritize shared decision-making and dignity preservation.^70^ Emerging evidence substantiates that such integrated approaches significantly mitigate emotional burden in cancer populations when implemented through timely, tailored interventions.^71^ To effectively modulate SCN trajectories, we propose a paradigm shift toward precision-supportive oncology incorporating three key elements: 1) implementing longitudinal distress tracking systems using ecological momentary assessment technologies, 2) developing machine learning algorithms to predict critical intervention timepoints, and 3) integrating mental health specialists into oncology care teams for real-time distress management. This tripartite model could disrupt the observed psychological-SCN feedback loops while maintaining fidelity to patient-centered care principles.

#### Social support

It is found that social support has a significant impact on the trajectory of the development of SCNs in cancer survivors, but there is some variation in results across studies, which may be related to differences in study populations, definitions of social support, or follow-up time points. Notably, family caring and main caregiver were also shown to have a significant impact on SCN trajectories. Family support is an important component of social support,^72^ and in the context of traditional Chinese culture, kinship is regarded as the most important source of family support. Besides, Chinese patients are more likely to share their concerns with family members than to seek help from professionals. Therefore, when family support is absent, patients’ need for supportive care shows a high level. It is recommended that family-based interventions be conducted in the future to enhance the importance of family care among family members.

#### Healthcare provision and community level factors

In addition, fewer studies have explored the impact of healthcare provision and community level factors on the SCNs trajectory, with only Qin finding that SCNs of rural patients tend to show a high level of decline (OR: 3.528; 95% CI: 1.083–11.490),^33^ which may be related to the fact that healthcare resources available in rural areas are relatively limited, and that patients tend to be more deficient in disease-related knowledge in the early stages of their illness. However, more studies have not found a correlation between place of residence and SCNs, and more evidence is needed to support the association.

### Implications for nursing practice and research

The findings from this study summarizes five types of SCNs, so dynamic intervention strategies can be developed in advance for patients in different trajectory groups based on this, providing precise interventions and reducing the workload of caregivers by reducing frequent assessments. Meanwhile, this study summarizes the factors affecting the SCNs trajectories of cancer survivors and a total of four main categories are classified using SEM, which provide a favorable basis for the early identification of populations with rapidly growing needs in clinical practice. In this regard, healthcare resources can be better allocated to high-risk populations, thus avoiding wasted resources. The identification of SCNs trajectories for cancer survivors transforms traditional ‘reactive’ care into ‘predictive’ management, enabling precision medicine through categorization and stratification. This not only improves patient experience and outcomes, but also provides a scientific basis for the efficient operation of the healthcare system, which is a key step towards value-based care. Future research could further explore the association between biopsychosocial factors and trajectories to refine intervention targets.

### Limitations

The exclusive focus on Chinese populations across all included studies introduces critical limitations regarding the generalizability of findings, constrained by the homogeneous geo-cultural representativeness encompassing genetic predispositions, sociocultural norms, and healthcare delivery paradigms. This geographic concentration precludes differentiation between universal biological mechanisms and culturally mediated expressions of SCNs in cancer survivors. Future research could expand the scope of the study to continue to explore the trajectory of SCNs in different regions of the world, compare the similarities and differences in the trajectory of SCNs between China and other regions, and identify culture-specific factors. Besides, we found that current research has examined fewer factors at the social support, healthcare provision and community level, and this systematic review only found family care, main carers, social support, and place of residence to be influential factors for the SCNs trajectory. However, these results were only validated in a few studies, and the effect of social support on the trajectory of SCNs presented different results across studies, which may be due to the heterogeneity of the studies. It is recommended that the predictive roles of social support, healthcare provision and community level factors on the trajectory of SCNs be further explored in the future based on the SEM to explore breakthroughs that are beneficial in meeting patients’ needs, thereby improving the physical and mental health and quality of life of cancer patients.^44^

## Conclusions

Our study provides the first systematic review of the trajectory of SCNs of cancer survivors. A total of ten studies are included and their results suggest that the trajectories of cancer survivor SCNs showed heterogeneity. However, there was heterogeneity in the SCNs trajectory categories regarding naming, number, level and trend, but this study grouped them into five categories by extracting similarities. For influencing factors of SCNs trajectory, it is mostly focused on demographic variables, clinical variables, and personal symptoms and the aspect of social support and healthcare provision and community level factors are gained less attention. Clinical caregivers should continue to focus on changes in cancer survivors’ SCNs over time and explore in greater depth the impact of social as well as policy support dimensions, with a view to developing rational programs to further improve the quality of life of cancer survivors.

## CRediT authorship contribution statement

**Kexin Li**: Conception, literature search, data extraction, data synthesis, and writing the original draft. **Shi Chen:** Writing, review and editing this article. **Xiaohui Dong:** Enhance the English quality of this article. **Xianying Lu, and Xinyu Chen**: Participate the process of quality evaluation and PRISMA checklist combed. **Ran Xu**: Provide ideas and revisions as well as a grant for this research. **Chaoming Hou and Jing Gao:** Conceptualization, supervision and review this article. All authors have read and approved the final manuscript.

## Ethics statement

Not required.

## Data availability statement

The authors confirm that the data supporting the findings of this study are available within the article and its supplementary materials.

## Declaration of generative AI and AI-assisted technologies in the writing process

No AI tools/services were used during the preparation of this work.

## Funding

This work was supported by Sichuan Medical Law Research Center [Grant No. 23YFYB008] and 10.13039/501100008402Chengdu University of Traditional Chinese Medicine, China (Grant No. MPRC2023050). The funders of the study had no role in study design, data collection, data extraction, data interpretation, or writing of this report.

## Declaration of competing interest

The authors declare no conflict of interest.
