# Supplementary material for: Group-based trajectory modeling for supportive care needs in Chinese cancer survivors: A systematic review
Source: Asia Pac J Oncol Nurs. 2025 Jun 6;12:100738. doi: 10.1016/j.apjon.2025.100738 (PMC12269420; doi:10.1016/j.apjon.2025.100738)
Supplement: Multimedia component 1 [file mmc1.docx]

**Supplementary Materials**

Appendix A: Search strategies of each database.

Appendix B: Evaluation criteria of JBI Checklist for Cohort Studies.

Appendix C: Methodological quality appraisal results based on the JBI critical appraisal tools for cohort studies for each study.

Appendix D: Methodological quality appraisal results and the details based on the Guidelines for Reporting on Latent Trajectory Studies (GRoLTS) Checklist for each study.

Appendix E: The details of statistical methods used by studies conducting group-based support care needs trajectory analysis.

**Appendix A**

| Appendix A | |
| --- | --- |
| Search strategies of each database. | |
| **Database** | **Index and keyword terms** |
| PubMed | #1 latent class analysis [MeSH Terms]  #2 ((((((((((((((((latent class growth model[Title/Abstract]) OR (latent class growth mixture model[Title/Abstract])) OR (latent class mixed model*[Title/Abstract])) OR (growth mixture model*[Title/Abstract])) OR (latent growth model[Title/Abstract])) OR (latent class growth analysis[Title/Abstract])) OR (latent class growth analyses[Title/Abstract])) OR (group based trajectory model*[Title/Abstract])) OR (group based trajectory analysis[Title/Abstract])) OR (group based trajectory analyses[Title/Abstract])) OR (group based model*[Title/Abstract])) OR (latent growth mixture model*[Title/Abstract])) OR (LCGA[Title/Abstract])) OR (LCGM[Title/Abstract])) OR (GMM[Title/Abstract])) OR (LGMM[Title/Abstract])) OR (LCMM[Title/Abstract])  #3 (trajectory [Title/Abstract]) OR (trajector*[Title/Abstract])  #4 (((((((((supportive care need*[Title/Abstract]) OR (supportive care[Title/Abstract])) OR (unmet need*[Title/Abstract])) OR (needs assessment[Title/Abstract])) OR (psychosocial care need*[Title/Abstract])) OR (social need*[Title/Abstract])) OR (emotional need*[Title/Abstract])) OR (physical need*[Title/Abstract])) OR (work need*[Title/Abstract])) OR (employment need*[Title/Abstract])  #5 (((cancer[Title/Abstract]) OR (neoplasm*[Title/Abstract])) OR (tumor[Title/Abstract])) OR (tumour[Title/Abstract])  #6 #1 OR #2 OR #3  #7 #4 AND #5 AND #6 |
| Web of Science | ((latent class analysis (Topic) or latent class growth model (Topic) or latent class growth mixture model (Topic) or latent class mixed model* (Topic) or growth mixture model* (Topic) or latent growth model (Topic) or latent class growth analysis (Topic) or latent class growth analyses (Topic) or group based trajectory model* (Topic) or group based trajectory analysis (Topic) or group based trajectory analyses (Topic) or group based model* (Topic) or latent growth mixture model* (Topic) or LCGA (Topic) or LCGM (Topic) or GMM (Topic) or LGMM (Topic) or LCMM (Topic)) OR (trajectory (Title) or trajectory* (Title) )) AND (supportive care need* (Title) or supportive care (Title) or unmet need* (Title) or needs assessment (Title) or psychosocial care need* (Title) or social need* (Title) or emotional need* (Title) or physical need* (Title) or work need* (Title) or employment need* (Title) ) AND (cancer (Title) or neoplasm* (Title) or tumor (Title) or tumour (Title)) |
| Scopus | (TITLE-ABS-KEY({supportive care need*}) OR ({unmet need*}) OR ({needs assessment}) OR ({psychosocial care need*}) OR ({social need*}) OR ({emotional need*}) OR ({physical need*}) OR ({work need*}) OR ({employment need*})) AND (TITLE-ABS-KEY({cancer}) OR ({neoplasm*}) OR ({tumo*r})) AND (TITLE-ABS-KEY({latent class analysis}) OR ({latent class growth model}) OR ({latent class growth mixture model}) OR ({latent class mixed model*}) OR ({growth mixture model*}) OR ({latent growth model}) OR ({latent class growth analysis}) OR ({latent class growth analyses}) OR ({group based trajectory model*}) OR ({group based trajectory analysis}) OR ({group based trajectory analyses}) OR ({group based model*}) OR ({latent growth mixture model*}) OR ({lcga}) OR ({lcgm}) OR ({gmm}) OR ({lgmm}) OR ({lcmm})) |
| EMBASE | ('latent class analysis':ab,ti OR 'latent class growth model':ab,ti OR 'latent class growth mixture model':ab,ti OR 'latent class mixed model*':ab,ti OR 'growth mixture model*':ab,ti OR 'latent growth model':ab,ti OR 'latent class growth analysis':ab,ti OR 'latent class growth analyses':ab,ti OR 'group based trajectory model*':ab,ti OR 'group based trajectory analysis':ab,ti OR 'group based trajectory analyses':ab,ti OR 'group based model*':ab,ti OR 'latent growth mixture model*':ab,ti OR lcga:ab,ti OR lcgm:ab,ti OR gmm:ab,ti OR lgmm:ab,ti OR lcmm:ab,ti) AND ('supportive care need*':ab,ti OR 'supportive care':ab,ti OR 'unmet need*':ab,ti OR 'needs assessment':ab,ti OR 'psychosocial care need*':ab,ti OR 'social need*':ab,ti OR 'emotional need*':ab,ti OR 'physical need*':ab,ti OR 'work need*':ab,ti OR 'employment need*':ab,ti) AND (cancer:ab,ti OR neoplasm*:ab,ti OR tumor:ab,ti OR tumour:ab,ti) |
| Cochrane library | ((trajectory):ti,ab,kw OR (trajectory*):ti,ab,kw) OR ((latent class analysis):ti,ab,kw OR (latent class growth model):ti,ab,kw OR (latent class growth mixture model):ti,ab,kw OR (latent class mixed model*):ti,ab,kw OR (growth mixture model*):ti,ab,kw) AND ((supportive care need*):ti,ab,kw OR (supportive care):ti,ab,kw OR (unmet need*):ti,ab,kw OR (needs assessment):ti,ab,kw) AND ((cancer):ti,ab,kw OR (neoplasm*):ti,ab,kw OR (tumor):ti,ab,kw OR (tumour):ti,ab,kw) |
| CINAHL | ((AB latent class analysis OR AB latent class growth model OR AB latent class growth mixture model OR AB latent class mixed model* OR AB growth mixture model* OR AB latent growth model OR AB latent class growth analysis OR AB latent class growth analyses OR AB group based trajectory model* OR AB group based trajectory analysis OR AB group based trajectory analyses OR AB group based model*、latent growth mixture model* ) OR (TI trajectory* OR TI trajectory )) AND (TI supportive care need* OR TI supportive care OR TI unmet need* OR TI needs assessment OR TI psychosocial care need* OR TI social need* OR TI emotional need* OR TI physical need* OR TI work need* OR TI employment need* ) AND (TI cancer OR TI neoplasm* OR TI tumor OR TI tumour ) |
| CNKI | 主题：（支持性照护需求 OR 支持性照顾需求 OR 支持性护理需求）AND (轨迹 OR 纵向研究) AND (癌症) |
| Wanfang database | 主题：(支持性照护需求 OR 支持性照顾需求 OR 支持性护理需求) AND (轨迹 OR 纵向研究) AND (癌症) |
| Vip database | 题名或关键词：（支持性照护需求 OR 支持性照顾需求 OR 支持性护理需求）AND (轨迹 OR 纵向研究) AND (癌症) |
| Sinomed | 常用字段：（支持性照护需求 OR 支持性照顾需求 OR 支持性护理需求）AND (轨迹 OR 纵向研究) AND (癌症) |

**Appendix B**

| Appendix B | |
| --- | --- |
| Evaluation criteria of JBI Checklist for Cohort Studies. | |
| **Item** | **Specific criteria for determining ‘Yes’** |
| 1. Were the two groups similar and recruited from the same population? | The two groups selected for comparison should be as similar as possible in all characteristics except for their exposure status, relevant to the study in question. The authors should provide clear inclusion and exclusion criteria that they developed prior to recruitment of the study participants. |
| 2. Were the exposures measured similarly to assign people to both exposed and unexposed groups? | The exposure measures should be clearly defined and described in detail. |
| 3. Was the exposure measured in a valid and reliable way? | The study should clearly describe the method of measurement of exposure. Assessing validity requires that a 'gold standard' is available to which the measure can be compared. The validity of exposure measurement usually relates to whether a current measure is appropriate or whether a measure of past exposure is needed. Reliability refers to the processes included in an epidemiological study to check repeatability of measurements of the exposures. These usually include intra-observer reliability and inter-observer reliability. |
| 4. Were confounding factors identified? | Typical confounders include baseline characteristics, prognostic factors, or concomitant exposures (e.g. smoking). |
| 5. Were strategies to deal with confounding factors stated? | Strategies to deal with effects of confounding factors may be dealt within the study design or in data analysis. By matching or stratifying sampling of participants, effects of confounding factors can be adjusted for. When dealing with adjustment in data analysis, assess the statistics used in the study. Most will be some form of multivariate regression analysis to account for the confounding factors measured. |
| 6. Were the groups/participants free of the outcome at the start of the study (or at the moment of exposure)? | The participants should be free of the outcomes of interest at the start of the study. Refer to the ‘methods’ section in the paper for this information, which is usually found in descriptions of participant/sample recruitment, definitions of variables, and/or inclusion/exclusion criteria. |
| 7. Were the outcomes measured in a valid and reliable way? | Read the methods section of the paper. If for e.g. lung cancer is assessed based on existing definitions or diagnostic criteria, then the answer to this question is likely to be yes. |
| 8. Was the follow up time reported and sufficient to be long enough for outcomes to occur? | The appropriate length of time for follow up will vary with the nature and characteristics of the population of interest and/or the intervention, disease or exposure. To estimate an appropriate duration of follow up, read across multiple papers and take note of the range for duration of follow up. The opinions of experts in clinical practice or clinical research may also assist in determining an appropriate duration of follow up. |
| 9. Was follow up complete, and if not, were the reasons to loss to follow up described and explored? | It is important in a cohort study that a greater percentage of people are followed up. As a general guideline, at least 80% of patients should be followed up. Generally, a dropout rate of 5% or less is considered insignificant. A rate of 20% or greater is considered to significantly impact on the validity of the study. Reporting of efforts to follow up participants that dropped out may be regarded as an indicator of a well conducted study. Look for clear and justifiable description of why people were left out, excluded, dropped out etc. If there is no clear description or a statement in the regards, this will be a 'No'. |
| 10. Were strategies to address incomplete follow up utilized? | Participants with unequal follow up periods must be taken into account in the analysis, which should be adjusted to allow for differences in length of follow up periods. This is usually done by calculating rates which use person-years at risk, i.e. considering time in the denominator. |
| 11. Was appropriate statistical analysis used? | As with any consideration of statistical analysis, consideration should be given to whether there was a more appropriate alternate statistical method that could have been used. The methods section of cohort studies should be detailed enough for reviewers to identify which analytical techniques were used (in particular, regression or stratification) and how specific confounders were measured. Additionally, it is also important to assess the appropriateness of the analytical strategy in terms of the assumptions associated with the approach as differing methods of analysis are based on differing assumptions about the data and how it will respond. |

**Appendix C**

| Appendix C | | | | | | | | | | |
| --- | --- | --- | --- | --- | --- | --- | --- | --- | --- | --- |
| The Joanna Briggs Institute (JBI) critical appraisal checklist for Cohort Studies. | | | | | | | | | | |
| **Item** | **Qin, 2022** | **Wang, 2022** | **Xiao, 2022** | **Wang, 2023** | **Yen et al, 2023** | **Liu et al, 2023** | **Yan et al, 2024** | **Zhang et al, 2024** | **Zhu et al, 2024** | **Zhou et al, 2024** |
| 1 | NA | NA | NA | NA | NA | NA | NA | NA | NA | NA |
| 2 | NA | NA | NA | NA | NA | NA | NA | NA | NA | NA |
| 3 | Yes | Yes | Yes | Yes | Yes | Yes | Yes | Yes | Yes | Yes |
| 4 | Yes | Yes | Yes | Yes | Yes | Yes | Yes | Yes | Yes | Yes |
| 5 | Yes | No | Yes | Yes | No | Yes | Yes | Yes | Yes | Yes |
| 6 | NA | NA | NA | NA | NA | NA | NA | NA | NA | NA |
| 7 | Yes | Yes | Yes | Yes | Yes | Yes | Yes | Yes | Yes | Yes |
| 8 | Yes | Yes | Yes | Yes | Yes | Yes | Yes | Yes | Yes | Yes |
| 9 | Yes | Yes | Yes | Yes | Yes | Yes | Yes | Yes | Yes | Yes |
| 10 | Yes | No | No | Yes | No | No | No | No | No | No |
| 11 | Yes | No | Yes | Yes | No | Yes | Yes | Yes | Yes | Yes |
| Proportion of yes | 72.7% | 45.5% | 63.6% | 72.7% | 45.5% | 63.6% | 63.6% | 63.6% | 63.6% | 63.6% |
| N/A, not applicable. **Details of items:** 1. Were the two groups similar and recruited from the same population? 2. Were the exposures measured similarly to assign people to both exposed and unexposed groups? 3. Was the exposure measured in a valid and reliable way? 4. Were confounding factors identified? 5. Were strategies to deal with confounding factors stated? 6. Were the groups/participants free of the outcome at the start of the study (or at the moment of exposure)? 7. Were the outcomes measured in a valid and reliable way? 8. Was the follow up time reported and sufficient to be long enough for outcomes to occur? 9. Was follow up complete, and if not, were the reasons to loss to follow up described and explored? 10. Were strategies to address incomplete follow up utilized? 11. Was appropriate statistical analysis used? **Quality cutoffs for research:** A percentage of “yes” greater than 70% is considered high quality, between 70% and 50% is moderate quality, and less than 50% is low quality. | | | | | | | | | | |

**Appendix D**

| Appendix D | | | | | | | | | | | |
| --- | --- | --- | --- | --- | --- | --- | --- | --- | --- | --- | --- |
| The Guidelines for Reporting on Latent Trajectory Studies (GRoLTS) Checklist. | | | | | | | | | | | |
| **Item** | **Qin, 2022** | **Wang, 2022** | **Xiao, 2022** | **Wang, 2023** | **Yen et al, 2023** | **Liu et al, 2023** | **Yan et al, 2024** | **Zhang et al, 2024** | **Zhu et al, 2024** | **Zhou et al, 2024** | **%** |
| 1 | 1 | 1 | 1 | 1 | 1 | 1 | 1 | 1 | 1 | 1 | 100% |
| 2 | 0 | 0 | 0 | 0 | 0 | 0 | 0 | 0 | 0 | 0 | 0% |
| 3a | 0 | 0 | 0 | 0 | 0 | 0 | 0 | 0 | 0 | 0 | 0% |
| 3b | 0 | 0 | 0 | 0 | 0 | 0 | 0 | 0 | 0 | 0 | 0% |
| 3c | 1 | 0 | 0 | 1 | 0 | 0 | 0 | 0 | 0 | 0 | 20% |
| 4 | 1 | 0 | 1 | 0 | 0 | 0 | 1 | 1 | 1 | 0 | 50% |
| 5 | 1 | 1 | 1 | 1 | 1 | 1 | 1 | 1 | 1 | 1 | 100% |
| 6a | 1 | 1 | 1 | 1 | 1 | 1 | 1 | 1 | 1 | 1 | 100% |
| 6b | 0 | 0 | 0 | 0 | 0 | 0 | 0 | 0 | 0 | 0 | 0% |
| 7 | 0 | 0 | 1 | 0 | 1 | 0 | 0 | 0 | 0 | 1 | 30% |
| 8 | 1 | 1 | 1 | 1 | 1 | 1 | 1 | 1 | 1 | 1 | 100% |
| 9 | 0 | 0 | 0 | 0 | 0 | 0 | 0 | 0 | 0 | 0 | 0% |
| 10 | 1 | 1 | 1 | 1 | 1 | 1 | 1 | 1 | 1 | 1 | 100% |
| 11 | 1 | 1 | 1 | 1 | 0 | 1 | 1 | 1 | 1 | 1 | 90% |
| 12 | 1 | 1 | 1 | 1 | 1 | 1 | 1 | 1 | 1 | 1 | 100% |
| 13 | 1 | 1 | 1 | 1 | 0 | 1 | 1 | 1 | 1 | 1 | 90% |
| 14a | 1 | 0 | 1 | 1 | 1 | 1 | 1 | 1 | 0 | 1 | 80% |
| 14b | 0 | 0 | 0 | 0 | 0 | 0 | 0 | 0 | 0 | 0 | 0% |
| 14c | 0 | 0 | 1 | 0 | 0 | 0 | 0 | 0 | 0 | 0 | 10% |
| 15 | 1 | 0 | 1 | 1 | 0 | 1 | 1 | 1 | 1 | 1 | 80% |
| 16 | 0 | 0 | 0 | 0 | 1 | 0 | 0 | 0 | 0 | 0 | 10% |
| Total score | 12 | 8 | 13 | 11 | 9 | 10 | 11 | 11 | 10 | 11 | 10.60 |
| 1 point = reported, 0 point = not reported. LGMM, latent growth mixture model; LCGA, latent class growth analysis. **Details of items:** 1. Is the metric of time used in the statistical model reported? 2. Is information presented about the mean and variance of time within a wave? 3a. Is the missing data mechanism reported? 3b. Is a description provided of what variables are related to attrition/missing data? 3c. Is a description provided of how missing data in the analyses were dealt with? 4. Is information about the distribution of the observed variables included? 5. Is the software mentioned? 6a. Are alternative specifications of within-class heterogeneity considered (e.g., LGCA vs. LGMM) and clearly documented? If not, was sufficient justification provided as to eliminate certain specifications from consideration? 6b. Are alternative specifications of the between-class differences in variance–covariance matrix structure considered and clearly documented? If not, was sufficient justification provided as to eliminate certain specifications from consideration? 7. Are alternative shape/functional forms of the trajectories described? 8. If covariates have been used, can analyses still be replicated? 9. Is information reported about the number of random start values and final iterations included? 10. Are the model comparison (and selection) tools described from a statistical perspective? 11. Are the total number of fitted models reported, including a one-class solution? 12. Are the number of cases per class reported for each model (absolute sample size, or proportion)? 13. If classification of cases in a trajectory is the goal, is entropy reported? 14a. Is a plot included with the estimated mean trajectories of the final solution? 14b. Are plots included with the estimated mean trajectories for each model? 14c. Is a plot included of the combination of estimated means of the final model and the observed individual trajectories split out for each latent class? 15. Are characteristics of the final class solution numerically described (i.e., means, SD/SE, n, CI, etc.)? 16. Are the syntax files available (either in the appendix, supplementary materials, or from the authors)? | | | | | | | | | | | |

| Appendix D (continued) | | | | | | | | | | | | | | | | | | | | | |
| --- | --- | --- | --- | --- | --- | --- | --- | --- | --- | --- | --- | --- | --- | --- | --- | --- | --- | --- | --- | --- | --- |
| The details of the GRoLTS checklist for the included studies. | | | | | | | | | | | | | | | | | | | | | |
| **Item**  **Study** | **1** | **2** | **3a** | **3b** | **3c** | **4** | **5** | **6a** | **6b** | **7** | **8** | **9** | **10** | **11** | **12** | **13** | **14a** | **14b** | **14c** | **15** | **16** |
| Qin, 2022 | 3 months | NR | NR | NR | Those with more than 20% omissions were eliminated | normally distributed | Mplus 8.3 | GMM | NR | NR | Yes | NR | AIC, BIC, aBIC, Entropy, LMR-LRT, BLRT | Reporting 5 numbers of classes | Class 1: n=40; Class 2: n=43; Class 3: n=87; Class 4: n=45 | Entropy=0.996 | Yes | NR | NR | Yes | NR |
| Wang, 2022 | 9 months | NR | NR | NR | NR | NR | R-Project | LCGM | NR | NR | Yes | NR | AIC, BIC, aBIC, Entropy, BLRT | Reporting 6 numbers of classes | Class 1: n=99; Class 2: n=73; Class 3: n=13; Class 4: n=26; Class 5: n=15 | Entropy=0.711 | NR | NR | NR | NR | NR |
| Xiao, 2022 | 8 months | NR | NR | NR | NR | normally distributed | Mplus 8.4 | LCGA | NR | Based on polynomial functions that is linear, quadratic, and free | Yes | NR | AIC, BIC, aBIC, Entropy, LMR-LRT, BLRT | Reporting 4 numbers of classes | Class 1: n=87; Class 2: n=69 | Entropy=0.83 | Yes | NR | Yes | Yes | NR |
| Wang, 2023 | 6 months | NR | NR | NR | Holographic great likelihood estimation | NR | Mplus 8.0 | LCGM | NR | NR | Yes | NR | AIC, BIC, aBIC, Entropy, LMR-LRT, BLRT | Reporting 4 numbers of classes | Class 1: n=63; Class 2: n=12; Class 3: n=47 | Entropy=0.874 | Yes | NR | NR | Yes | NR |
| Yen et al, 2023 | 4 months | NR | NR | NR | NR | NR | SAS software Version 9.4 | GBTM | NR | Based on constant, linear, or quadrat patterns | Yes | NR | BIC | NR | Class 1: n=26; Class 2: n=31; Class 3: n=14 | NR | Yes | NR | NR | NR | Yes |
| Liu et al, 2023 | 3 months | NR | NR | NR | NR | NR | Mplus 7.0 | LCGM | NR | NR | Yes | NR | AIC, BIC, aBIC, Entropy, LMR-LRT, BLRT | Reporting 5 numbers of classes | Class 1: n=82; Class 2: n=54; Class 3: n=41 | Entropy=0.894 | Yes | NR | NR | Yes | NR |
| Yan et al, 2024 | 6 months | NR | NR | NR | NR | normally distributed | Mplus 7.1 | LCGM | NR | NR | Yes | NR | AIC, BIC, aBIC, Entropy, LMR-LRT, BLRT | Reporting 4 numbers of classes | Class 1: n=74; Class 2: n=58; Class 3: n=42 | Entropy=0.831 | Yes | NR | NR | Yes | NR |
| Zhang et al, 2024 | 6 months | NR | NR | NR | NR | normally distributed | Mplus 8.0 | LCGA | NR | NR | Yes | NR | AIC, BIC, aBIC, Entropy, LMR-LRT, BLRT | Reporting 5 numbers of classes | Class 1: n=51; Class 2: n=109; Class 3: n=89 | Entropy=0.847 | Yes | NR | NR | Yes | NR |
| Zhu et al, 2024 | 6 months | NR | NR | NR | NR | normally distributed | Mplus 8.3 | LCGM | NR | NR | Yes | NR | AIC, BIC, aBIC, Entropy, LMR-LRT, BLRT | Reporting 5 numbers of classes | Class 1: n=77; Class 2: n=69; Class 3: n=74 | Entropy=0.776 | NR | NR | NR | Yes | NR |
| Zhou et al, 2024 | 3 months | NR | NR | NR | NR | NR | Mplus 8.3 | GMM | NR | Based on linear pattern | Yes | NR | AIC, BIC, aBIC, Entropy, LMR-LRT, BLRT | Reporting 4 numbers of classes | Class 1: n=136; Class 2: n=43; Class 3: n=53 | Entropy=0.813 | Yes | NR | NR | Yes | NR |
| LGMM, latent growth mixture model; LCGA, latent class growth analysis. | | | | | | | | | | | | | | | | | | | | | |

**Appendix E**

| Appendix E | | | | | | | | | | |
| --- | --- | --- | --- | --- | --- | --- | --- | --- | --- | --- |
| Statistical methods used by studies conducting group-based support care needs trajectory analysis. | | | | | | | | | | |
| **Studies** | **Statistical method** | **Software** | **AIC** | **BIC** | **aBIC** | **Entropy** | **LMR-LRT** | **BLRT** | **Other** | **Missing Value Handling** |
| Qin, 2022 | GMM | Mplus 8.3 | √ | √ | √ | √ | √ | √ | NR | Those with more than 20% omissions were eliminated. |
| Wang, 2022 | LCGM | R-Project | √ | √ | √ | √ | NR | √ | NR | NR |
| Xiao, 2022 | LCGA | Mplus 8.4 | √ | √ | √ | √ | √ | √ | NR | NR |
| Wang, 2023 | LCGM | Mplus 8.0 | √ | √ | √ | √ | √ | √ | NR | Holographic great likelihood estimation |
| Yen et al, 2023 | GBTM | SAS software Version 9.4 | NR | √ | NR | NR | NR | NR | NR | NR |
| Liu et al, 2023 | LCGM | Mplus 7.0 | √ | √ | √ | √ | √ | √ | NR | NR |
| Yan et al, 2024 | LCGM | Mplus 7.1 | √ | √ | √ | √ | √ | √ | NR | NR |
| Zhang et al, 2024 | LCGA | Mplus 8.0 | √ | √ | √ | √ | √ | √ | NR | NR |
| Zhu et al, 2024 | LCGM | Mplus 8.3 | √ | √ | √ | √ | √ | √ | NR | NR |
| Zhou et al, 2024 | GMM | Mplus 8.3 | √ | √ | √ | √ | √ | √ | NR | NR |
| NR, Not Report; AIC, Akaike Information Criterion; BIC, Bayesian Information Criterion; aBIC, adjusted Bayesian Information Criterion; SSBIC, sample size-adjusted Bayesian; SABIC, Sample-Size Adjusted Bayesian Information Criteria; LMR-LRT, Lo–Mendell–Rubin likelihood ratio test; BLRT, Bootstrap likelihood ratio Test; LGMM, latent growth mixture model; GMM, growth mixture model; LCGM, latent class growth model; LCGA, latent class growth analysis; GBTM, Group based trajectory modeling | | | | | | | | | | |
